# International Congress of Dermatology

**Published:** 1896-06

**Authors:** 


### INTERNATIONAL CONGRESS OF DERMATOLOGY.

The third Congress is to be held in London from the 4th to the 8th of August. Mr. Jonathan Hutchinson is the President. All information concerning the Congress can be obtained from the Secretary-General, Dr. J. J. Pringle, 23 Lower Seymour Street, London, W.

